# 

**Published:** 1897-07-10

**Authors:** 


					HLuv/i-<b "ir1 ui\i:, mcrcrurc GrTSuu uui^i xviu, ?oui,
CADBURY'S ft CADBURY'S
OOCOA m Ml COCOA
<?kii* HO ALKALIES USEr.
?-J?*(TUiASQflJr n EST SAN INF IBM ART
No.-563. Vol. XXII. [fSSfpi!] Saitoday,
July 10th, 1897.
[Subscription Terms,] pEJCE TWOPENCE.

				

